# An Insight into Current Treatment Strategies, Their Limitations, and Ongoing Developments in Vaccine Technologies against Herpes Simplex Infections

**DOI:** 10.3390/vaccines11020206

**Published:** 2023-01-17

**Authors:** Divya Sharma, Supriya Sharma, Natasha Akojwar, Ayusha Dondulkar, Nikhil Yenorkar, Deepti Pandita, Satyendra K. Prasad, Mahaveer Dhobi

**Affiliations:** 1 School of Pharmaceutical Sciences, Delhi Pharmaceutical Sciences and Research University, Sector-III, Pushp Vihar, Government of NCT of Delhi, New Delhi 110017, India; 2 Department of Pharmaceutical Sciences, Rashtrasant Tukadoji Maharaj Nagpur University, Nagpur 440033, India; 3 Delhi Institute of Pharmaceutical Sciences and Research, Delhi Pharmaceutical Sciences and Research University, Sector-III, Pushp Vihar, Government of NCT of Delhi, New Delhi 110017, India

**Keywords:** herpes simplex virus, herpes labialis, vaccine, antiviral, acyclovir, immune

## Abstract

Herpes simplex virus (HSV) infection, the most prevalent viral infection that typically lasts for a lifetime, is associated with frequent outbreaks of oral and genital lesions. Oral herpes infection is mainly associated with HSV-1 through oral contact, while genital herpes originates due to HSV-2 and is categorized under sexually transmitted diseases. Immunocompromised patients and children are more prone to HSV infection. Over the years, various attempts have been made to find potential targets for the prevention of HSV infection. Despite the global distress caused by HSV infections, there are no licensed prophylactic and therapeutic vaccines available on the market against HSV. Nevertheless, there are numerous promising candidates in the pre-clinical and clinical stages of study. The present review gives an overview of two herpes viruses, their history, and life cycle, and different treatments adopted presently against HSV infections and their associated limitations. Majorly, the review covers the recent investigations being carried out globally regarding various vaccine strategies against oral and genital herpes virus infections, together with the recent and advanced nanotechnological approaches for vaccine development. Consequently, it gives an insight to researchers as well as people from the health sector about the challenges and upcoming solutions associated with treatment and vaccine development against HSV infections.

## 1. Introduction

For a century, the term ‘herpes’ meaning to crawl or to creep has been used in the Greek medicine system. The term cold sore (herpes fibrils) was described by Roman physician Herodotus. Later, in 1736, the term genital herpes was coined by physician John Astruc, and in 1754 its first English translation appeared in his treatise on venereal disease. In the 18th century, herpes simplex virus (HSV) was denoted as a vocational disease of women as it was more prevalent in prostitutes [1]. It was observed in the late 19th century that the orolabial lesion was transferred to other people. According to Nahmias and Dawdle’s report presented in 1960, there are two types of antigenic HSV based on different sites of viral infection. The two serotypes of HSV, HSV-1 and HSV-2, belong to the family Herpesviridae. The three subfamilies of the Herpesviridae family are alpha-herpesviruses, beta-herpesviruses, and gamma-herpesviruses. Although all members of the Herpesviridae family can enter latency (the state of being dormant within the host cell), the cells in which they do so differ. HSV-1 and HSV-2 are members of the alpha-herpesvirus subfamily, have a short replication cycle, and can infect a variety of hosts [2].

The most common diagnostic indication of HSV infection is herpes labialis and herpes genitalis. At the beginning of the infection, the development of vesicular sores/ulcers in the oro-facial and genital regions may appear, slowly drying out into crusts that last up to 10–14 days [3,4]. Despite antiviral therapy, HSV-1 and HSV-2 can cause serious illnesses in immunocompromised children and adults, such as encephalitis with complications [5].

Synthetic medications for the treatment of HSV infection were developed in 1960, particularly drugs that restricted viral replication. First, an antiviral drug that was successfully administered systemically for HSV treatment included vidarabine as it showed better therapeutic efficacy and less toxicity [6]. Currently, acyclovir (ACV), a synthetic acyclic guanosine analogue is frequently used to treat HSV-1 and HSV-2 infections because of its accessibility, tolerability, and effectiveness [7,8,9] Some other Food and Drug Administration (FDA)-approved antiviral medications including penciclovir, famciclovir, ganciclovir, valacyclovir, cidofovir, and foscarnet are currently being used for the treatment of skin lesions caused by HSV-1 and HSV-2. However, the limited efficacy of these antiviral medications is a major concern, as they only shorten the recovery period of infection by usually 1–2 days [10,11,12]. In addition, viral resistance and side effects of these antiviral therapies pose challenges in the treatment of HSV infection [13]. It has also been reported that the effectiveness of these therapies is not noticeable for some people [14]. Apart from synthetic medicines, numerous attempts have been made to develop vaccines at a global level to manage HSV infection. Considering the genetic similarities between HSV-1 and HSV-2, establishing a vaccine that shows success against one kind of the HSV might be beneficial for the other type. However, the development of vaccines to combat the virus has proven extremely difficult because of viral latency and HSV immune evasion [15,16]. Thus, until now, no vaccine has been authorized for the use in humans. As per clinical and pre-clinical data, it has been observed that a vaccination strategy which increases effector memory T cell responses will be required for developing a potent HSV vaccine [17]. In the present review, we highlight the current treatment strategies, their limitations, and ongoing clinical studies for the development of a vaccine for managing HSV infections. The current review provides in-depth understanding about various vaccination strategies to develop an effective, yet safe vaccine for HSV infection.

## 2. Herpes Simplex Virus (HSV): An Overview

The Herpesviridae family includes HSVs, which are double-stranded DNA viruses having icosahedral symmetry. HSV-1 primarily spreads through oral-to-oral contact and produces an infection in or around the mouth (oral herpes), but it can also result in genital herpes. Genital herpes is mostly caused by the sexually transmitted HSV-2 virus [18]. WHO estimates that 3.7 billion people worldwide under the age of 50 have HSV-1 infection (67%) and 491 million people (13%) between the ages of 15 and 49 have HSV-2 infection [19]. HSV-1 mostly causes oral, labial, and possibly facial lesions. This primary infection is more serious and can cause ulcerative, painful stomatitis in children, and is associated with fever, malnutrition, and severe oral mucosal swelling [20]. Almost 30% of individuals have been affected due to repetitive orolabial infection with serologic evidence of HSV-1 and 40% people encounter frequent infections every year. Viral reactivation is spontaneous but has been linked to stress (either physical or mental), fever, UV exposure, tissue injury, and immunological suppression [21]. Recently, it has been stated that genital herpes is caused by both HSV-1 and HSV-2 mostly in industrialized nations [22,23]. Primary genital herpes develops in the form of papules and macules which are followed by vesicles, pustules, and ulcers. The non-primary genital infection is less severe than the primary infection as it gets healed within two weeks and has minor lesions, less pain, and a lower likelihood of negative consequences [21]. In genital herpes, clinical symptoms include painful genital vesicles and ulcers followed by inguinal adenopathy and systemic flu-like condition [24]. In genital infection, females are more susceptible to the formation of ulcers than males, and the progression of lesions is like that of oral herpes, where males experience a longer vesicular phase [25]. Both HSV-1 and HSV-2 use the same mechanism to bind, fuse, and proliferate from cell to cell [26]. They exhibit tremendous structural analogy, sharing about 82% of the amino acid sequence [27]. Since few studies have been reported for HSV-2, most of the concepts developed for HSV-1 are applied to HSV-2 owing to their high structural similarity [28]. HSV consists of a distinctive four-layered structure: a DNA core, capsid, tegument, and lipid membrane [29]. The electron-dense core of both HSV viruses consists of linear DNA made up of two components, L (long) and S (short), that are covalently linked to each other in addition to unique sequences, U_l_ (unique long) and U_s_ (unique short) [30,31]. The genome length of HSV-1 and HSV-2 are approximately 152, kbp and 154, kbp, respectively. The genomic sequence of both HSV-1 and HSV-2 are closely related with only a major difference in the Us region, which is 1349 bp longer in HSV-2 [32]. The UL components of HSV-1 and HSV-2 genomes have the highest similarities, while the inverted repeats that bind UL share the least similarities (TRL and IRL). The nucleotide sequence of HSV-1 and HSV-2 reveals that the similarity is greatest across UL, except for UL 42 through UL 44 and UL 49. Most of the peaks correspond to protein-coding areas, and the valleys to intergenic regions. The peak of similarity is in the region encoding ICP0 (RL2), whereas the L component repeats are substantially less conserved. The ICP4 gene (RS1) contains the S component repeats, which is the most conserved region. Overall, US is not as well-conserved as UL. This region contains the US 4 (gG) gene, which is about 1500 bp longer in HSV-2 than in HSV-1 and constitutes the region with the highest divergence between HSV-1 and HSV-2 [33].

**Figure 1:**
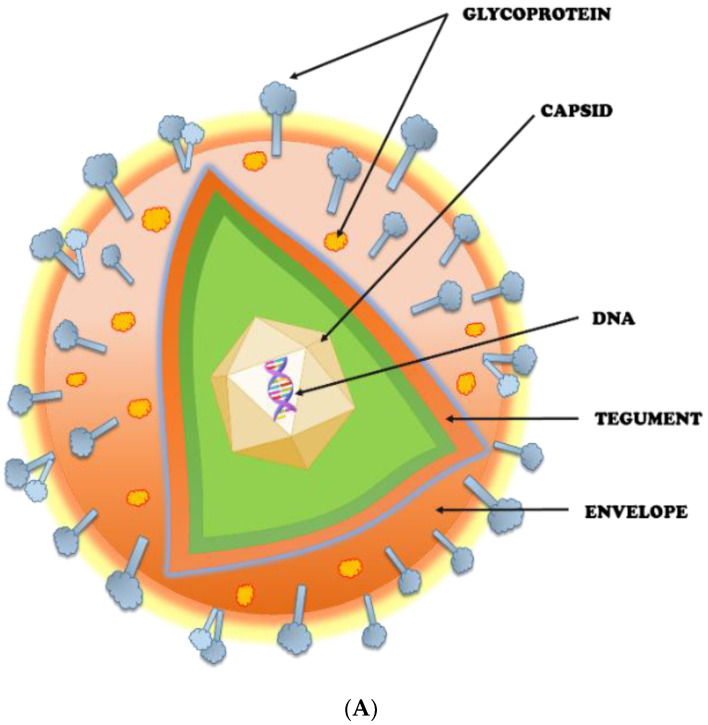

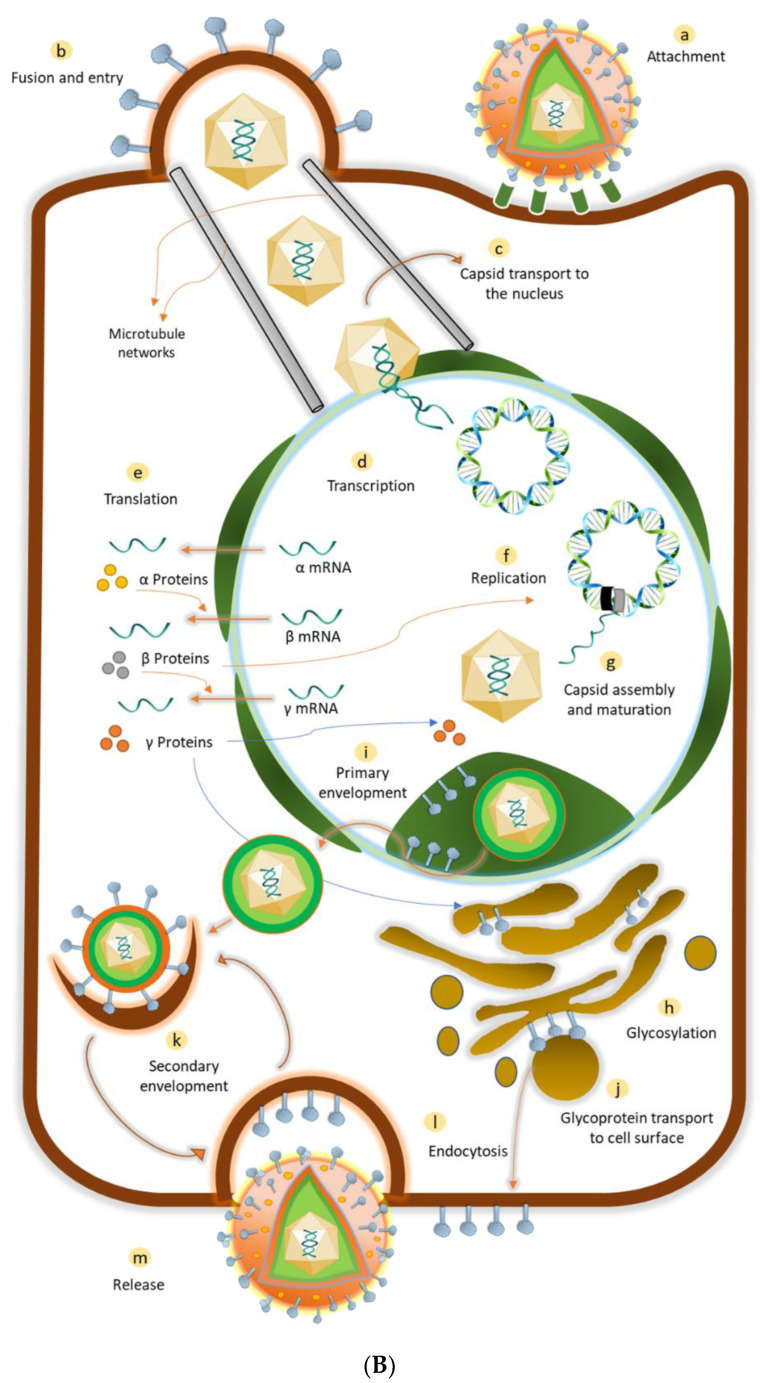
(**A**) Structure of herpes virus; (**B**) Life cycle of herpes virus.

The capsid of HSV is a well-defined icosahedral made of four proteins, the major one being VP5 (149 kDa, 1374 amino acids) [34]. It helps to guard the functionality of viral DNA when outside the host cell and to initiate the infection process [35]. The tegument is an amorphous protein, whose function includes modulation of viral and host protein expression, targeting viral components, and assembly of virus [36]. Approximately twenty-two viral proteins are found in the tegument underneath the envelope [37]. The important HSV-1 teguments include pUS3, pUL13, pUL36, and pUL37 [36], and HSV-2 teguments include VP11/12, VP13/14, and VP22 [38]. This entire structure is enclosed in a glycoprotein-embedded lipid bilayer envelope. HSV has about 85 genes, out of which 12 genes code for glycoproteins [39]. These glycoproteins mediate the attachment and penetration of the virus into the host cell. These 12 different types of glycoproteins (gB, gC, gD, gE, gG, gH, gI, gJ, gK, gL, gM, and gN) are present in the envelope, out of which four glycoproteins have been associated with the process of viral entry, viz., gB, gD, gH, and gL, commonly called core fusion machinery. Figure 1: A and B represent the structure and life cycle of a typical herpesvirus. Generally, HSV-1 mostly spreads by contact with sores, saliva, or surfaces in or near the mouth that have been exposed to the virus. HSV-2 is the virus that typically causes genital HSV infections, while HSV-1 is responsible for an increasing percentage of cases. Compared to HSV-2 infections, genital HSV-1 infections are often less severe and less likely to reoccur. HSV-2 mostly spreads during intercourse by contact with the anal or genital surfaces, skin, lesions, or fluids of an infected person. HSV-2 can even spread if the skin seems normal and frequently does so without causing any symptoms [40,41]. HSV ensures its survival by sustaining a lifelong invasion of its host by establishing latency in peripheral neurons. HSV penetrates through breaches in the skin surface or mucosa by direct contact, rapidly establishing acute infections in the skin while also gaining access to the nerves that innervate the infection site. The virus invades peripheral neuron cell bodies, which are gathered in sensory and autonomic ganglia, by parasitizing axonal transport [42,43]. There are several outcomes for these neurons, ranging from productive infection at one end of the spectrum to the rapid development of latency at the other [44,45]. Furthermore, some acutely infected neurons block this function and live to join the population of latently infected neurons [46,47]. Latently infected neurons serve as a reservoir of virus for further episodes of productive infection if reactivation takes place after active HSV has been eradicated. A well-known cascade of lytic gene expression is linked to the active stage of productive or lytic infection. Contrarily, the latent state is typically thought to include strict viral gene regulation, and HSV has been seen as a model for viral latency in general, and in fact served as the inspiration for the early treatment of other viruses [48].

### 2.1. Potential Targets for HSV Prevention

There have been major attempts over the last several years to build up a variety of methodologies for the discovery of prospective new antiviral medications (enlisted in Table 1). The discovery and confirmation of viral targets have been the topic of several studies employing several techniques. One of the major targets is DNA polymerase; the viral DNA polymerase from HSV-1-infected cells is a heterodimer of two catalytic subunits encoded by UL30 and UL42. The DNA polymerase enzyme is inhibited by drugs like 4-hydroxy-quinoline-3-carboxamide and 8-hydroxyquinoline [49,50,51]. Since the interaction between UL30 and UL42 is essential for viral replication, its disruption suggests a potential target for developing novel antiviral therapeutics [52,53]. At least five of the HSV-1 viral envelope glycoproteins-gC, gB, gD, gH, and gL, along with host-cell membrane receptors, including herpes virus entry mediator (HVEM), nectin-1, nectin-2, and extracellular glycosaminoglycans, are required for HSV attachment and penetration into the cell [54]. One strategy is direct inhibition of viral attachment factors by using substances such as PRO 2000, polymethylene hydroquinone sulfonate, and cellulose sulphate that stop the virus from invading or replicating by building a stable complex with these proteins [55,56]. Three viral proteins, UL5, UL8, and UL52, which make up the enzyme helicase primase, are involved in the synthesis of DNA by viral DNA polymerase. Therapeutic medications such as 2-amino-thiazole and thiazolylsulfonamide decrease helicase primase production and limit HSV replication [57]. Ribonucleotide reductase (RR), which transforms ribonucleoside diphosphate into deoxyribonucleotides and is required for DNA synthesis, is another target. The interaction of two dimeric subunits, designated as RR1 and RR2, is essential for enzymatic activity. The interaction of the two subunits is selectively inhibited by therapeutic drugs like peptidomimetic inhibitors, which stop replication of HSV [58,59]. Interaction with viral particle, cellular receptor, or both to prevent virus entry is an alternate strategy since virus–host interactions are dynamic and complicated processes. Targeting certain genes to prevent viral infections seems possible with the advent of specialised genome editing tools, such as ZFNs (zinc-finger nucleases), TALENs (transcription activator-like endonucleases), and the CRISPR/Cas9 (CRISPR-associated protein 9) system [60]. A possible therapy for viral genome replication at the RNA level is synthetic siRNA. Targeting the viral DNA polymerase enzyme pUL54, kinase pUL97, and immediate-early genes UL123 and UL122 by siRNAs has been demonstrated in in vitro tests to successfully limit HCMV infection [61]. In HSV-1-infected cells, the polyamine biosynthesis pathway is still active, and S-adenosyl methionine decarboxylase (SAMDC) and ornithine decarboxylase expression is increased [62]. This supports the idea that polyamines play a part in the HSV-1 replication cycle [63,64]. The in vitro replication of HSV-1 and HSV-2 laboratory strains, as well as clinical mutant isolates that are resistant to traditional antiviral medications like ACV and foscarnet, is also inhibited by suppression of SAMDC activity [64]. SAMDC and other important polyamine pathway enzymes thus provide viable targets for anti-HSV therapy. Recent research has demonstrated that certain cyclin-dependent kinases (CDKS) are necessary for HSV replication. Pharmacological CDK inhibitors (PCIs) can prevent HSV-1 infection through several different methods. The PCIs stop viral DNA synthesis and immediate-early and early gene expression activation. The viral proteins ICP4 and ICP0 with ability to phosphorylate are disrupted by roscovitine [65,66]. By interfering with nuclear factor-κB (NF-κB) signalling at an early stage of virus infection, toll-like receptor (TLR) inhibitors greatly reduce the ability of viruses to replicate. Treatment with five adjacent guanosine residues (G-ODN) 2 h before infection suppressed TLR9 signalling and NF- κB activity and markedly decreased the production of lytic virus in herpes-susceptible cells. Additionally, the TLR9 inhibitory action of CpG oligonucleotide has been linked to downregulation of essential immediate early HSV proteins, reduced viral entry and adhesion, virucide activity, and reduced virus multiplication [67].

### 2.2. Interplay of HSV and Immune System

The immune response generated against these viruses is complex and involves an interplay between innate and adaptive immune pathways. The host–pathogen interaction could lead to a diseased state. The pathogen’s virulent factors (encoded proteins) may aid it in overcoming the host’s immune defences. The detection of HSV-1 and the induction of interferon type 1 and inflammatory cytokines have been reported to be mediated by toll-like receptors 2 and 3 [68]. TLR-3 expression and interferon type 1 production have both been shown to be inhibited by HSV-1 tegument kinase US3 [69]. Additionally, the HSV-1-infected cell polypeptide 0 (ICP0) significantly lowers the amounts of myD88, a crucial adaptor protein of the TLR pathway, in the cells [70]. It has been widely reviewed how this ICP0 protein contributes to HSV-1 infection [71]. Furthermore, NF-κB activation of genes related to an inflammatory response has been shown to be inhibited by HSV [72]. However, HSV-1 _γ1_34.5 protein suppresses NF-κB expression in CD8+ dendritic cells [73]. In addition, it has been noted that the tegument protein VHS inhibits viral replication-independent NF-κB expression [74]. The TNF-α dependent activation of NF-κB is prevented by the HSV-1 protein UL42 [72]. It was reported that UL42 binds to the p65 and p50 subunits of NF-κB and prevents their translocation into the nucleus. As a result, the transcription of genes implicated in inflammatory responses is suppressed. The RLR cytosolic signalling pathway can trigger other cytokines such as type I interferon. Like this, the NF-κB is stimulated by the TLR2/TIR/MyD88/Mal signalling pathway, which facilitates the production of pro-inflammatory cytokines such as interleukins 6, 8, and 12 [75]. IRF3 and IRF7 are activated by TLR2/TIR/MyD88 signalling, which stimulates the synthesis of interferon-alpha and beta [76]. In addition, CD8+ TRM, a subtype of memory lymphocyte that dwells in non-lymphoid tissues [77], has been shown to activate the adaptive immune response in HSV-1 infection [78] via IFN-Y and granzyme B effectors. Following initial infection, it has been observed that CD4+ cells prevent HSV-1 infection and eradicate it from vaginal infection sites [79]. The depletion of CD4+ cells enhanced the vulnerability of mice to HSV-1 infection [80], and CD4+ CD25+ cells have been implicated in the HSV-1 response [81]. There has been a thorough study of the function of adaptive immune cells in HSV-1 infection [82]. Studies have reported that HSV inhibits the process of autophagy via the process of phosphorylation, using HSV protein Us3 ser/thr kinase of two factors involved in autophagy, namely, ULK1 and beclin1 [83]. Other suggested mechanisms exerted by HSV for modulating autophagy include downregulation of adaptor optineurin protein, autophagy adaptor protein sequestosome, and mitophagy, which are important mediators for decreasing the viral load [84]. Furthermore, PML-NBs, promyelocytic leukaemia nuclear bodies, form structures termed as viral DNA-containing PML-NBs that restrict HSV genetic expression and its subsequent replication. However, this mechanism to restrict HSV gene expression is counteracted by the ICP0 protein produced by the virus [85]. Inhibition of this apoptosis by HSV is significant for the reactivation of the virus from latency. Us3, which is a serine/threonine kinase, inhibits bad-induced apoptosis, whereas Us5 is a glycosylated J protein that inhibits fas-mediated apoptosis [86]. HSV moves into and out of the neuronal cell body throughout its life cycle within neuronal processes. HSV first affects the mucous membranes of the mouth or eyes before moving on to invade sensory nerve terminals. Retrograde transport allows HSV to go from the neural process back to the cell body, where it either enters latency or starts to multiply. Once replicated, HSV uses an anterograde transport mechanism, i.e., transit from the cell body, towards the mucosal membrane to disseminate the virus within the host as well as to other hosts. The virus has successfully spread through this cycle of retrograde transit, latency, replication, and anterograde transport. Kinesin1 proteins such as KIF5A, 5B, and 5C play a significant role in this anterograde transport mechanism. Targeting this transport mechanism can prove to be a potential therapeutic intervention [87]. The proteins expressed by HSV are known to hamper the translocation of sting protein from the endoplasmic reticulum to the Golgi apparatus, which is an essential mechanism for cellular immunity. This results in decreased levels of interferon responses [88]. Moreover, IFIT3 is an antiviral factor produced by the host that limits the replication of viral nucleic acid. UL41, a tegument protein, is reported to disrupt the IFIT3 protein activity [89]. Downregulating the CD1d expression is yet another mechanism employed by the virus to evade the immune response [90]. Peroxisomes play an important role in signalling pathways against invading pathogens. Another study revealed that VP16, a viral tegument protein, dampens the early antiviral immunity signalling from peroxisomes [91]. Finally, microRNAs (miRNA) are non-coding ribonucleic acids of 20–24 nucleotide short sequences that are responsible for post-transcriptional regulation. The decreased lytic gene expression through latency, increased viral replication, and antagonistic effect on innate and intrinsic immunity are among the hypothesised roles of HSV-1 miRNAs. Understanding the regulation of miRNA expression is crucial since miRNAs have been hypothesised to affect the switch from lytic to latent stage of HSV. Six miRNAs, namely, miR-592, miR-1245b-5p, miR-150, miR-342-5p, miR-1245b-3p, and miR-124, are known to reduce the expression of antiviral protein produced by the host cell as well as reduce viral recognition and clearance by natural killer cells [92].

## 3. Therapeutic Strategies Available for Herpes Infections

**Figure 2:**
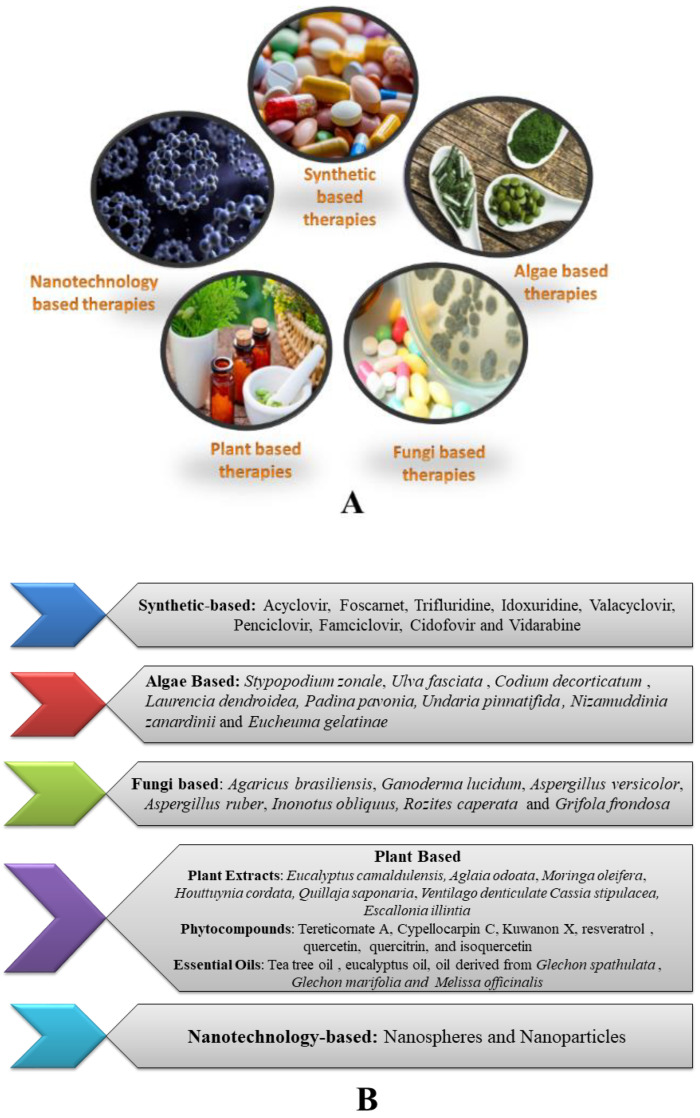
(**A**): Various therapeutic strategies for the treatment of HSV infection; (**B**): Examples under different categories of HSV therapeutic strategies.

Numerous therapeutic strategies are available to combat infection caused by HSV-1 and HSV-2. These strategies include synthetic drugs, plant extracts, plant-derived compounds, algae-based therapies, fungi-based therapies, and nanotechnology-based therapies. Examples of each category are illustrated in Figure 2. These various therapeutic regimens act through different mechanisms and help to reduce severe damage caused by the herpes virus [93]. The majority of formulations made from natural substances and synthetic medicines are administered to reduce symptoms caused by HSV-1 and HSV-2. [94].

### 3.1. Synthetic Therapeutics

Modern medicines or antivirals are currently available on the market to treat HSV-1 and HSV-2 infections. Acyclic nucleoside and nucleotide analogues are a large class of antivirals that have been licenced for the treatment of HSV-1 and HSV-2 infections [40,41]. These antivirals prevent viral DNA polymerase from extending the viral genome during replication (UL30). ACV is the most common first-line treatment drug used to treat herpes caused by HSV-1 and HSV-2. ACV must be phosphorylated into acyclovir triphosphate in order to exert its antiviral action intracellularly. This mechanism is accomplished by the viral thymidine kinase (TK, UL23 gene), which catalyses the conversion of acyclovir into acyclovir monophosphate, thus decreasing its escape from the cell and thereby its levels within the infected cells [9,93]. ACV undergoes further phosphorylation, and on reaching its triphosphate form, it functions as a substrate for the viral DNA polymerase, thus inhibiting DNA synthesis [95]. ACV is not the only treatment option for herpes simplex skin lesions; additional options include valacyclovir, penciclovir, and famciclovir, which are also utilised frequently and are first-line medications for treating oral and genital herpetic lesions caused by HSV-1 and HSV-2 [96]. These drugs, which share a similar mode of action as ACV, are nucleic acid analogues which work by interfering with the viral DNA polymerase functioning [95]. When applied topically, these drugs alleviate herpetic lesions and accompanying discomfort in around 1–3 days as compared to untreated groups. These drugs differ from one another primarily in their bioavailability, half-life, and dose [97]. Valacyclovir is an ACV prodrug that has improved intestinal absorption [98]. Penciclovir was produced later with the goal of being phosphorylated more quickly than ACV and, as a result, achieving a longer half-life than ACV. Famciclovir is a prodrug that converts into penciclovir and has a higher oral bioavailability [99]. The efficacy of these medications in treating skin lesions brought on by HSV infections has also been sparked by their positive clinical results [14,100]. Additionally, valacyclovir has been licenced for the treatment of HSV-1 and HSV-2 infections and clinical manifestations caused by HSV-1 and HSV-2, including cold sores and recurrent genital herpes [101]. Moreover, famciclovir is authorised for the treatment of genital herpes and orolabial herpes [102].

Nucleoside analogues including idoxuridine and vidarabine are very popular to show effective results against HSV. Idoxuridine is a pyrimidine analogue used for the treatment of HSV infection [103]. It is primarily applied topically as an ointment to treat epithelial keratitis brought on by HSV-1 infection of the corneal epithelium [104]. Its weak hydrosolubility and toxicity to the corneal epithelium of the eye, however, cast doubt on its usefulness, and it is now being replaced by other more efficient, more tolerable, and less toxic drugs [103]. Vidarabine, on the other hand, is a purine analogue that has less adverse effects than idoxuridine but is also sparingly soluble and can only be used in topical formulations, making it less effective than other drugs that are already on the market. Similarly, on the other hand, trifluridine is routinely used topically to treat herpetic keratitis. This medication was given FDA approval in 1980 for the treatment of HSV-related keratitis as a 1% solution, and it is currently one of the most widely used topical antivirals for this condition in the United States, with significant efficacy documented [105]. Brivudine is also a pyrimidine analogue that functions as a prodrug and is phosphorylated by viral thymidine kinase exclusively to target the viral DNA polymerase. It has been demonstrated that this drug is effective in treating HSV-1 infection and is currently used mostly in treating VZV infections in various nations [103]. It has been observed that the nucleic acid analogue ganciclovir, which is used to treat cytomegalovirus (CMV) infection, especially in immunocompromised individuals with systemic and ocular infections, is also effective against HSV-1 and HSV-2 [105,106]. 

The acyclic nucleotide and pyrophosphate analogues, namely, foscarnet and cidofovir, are alternative options for the treatment of HSV. Foscarnet usually blocks viral DNA polymerase to activate polymerase activity [106,107], whereas cidofovir has been shown to be effective in vitro against HSV-1 or HSV-2 isolates that are resistant to acyclovir [108]. Immunosuppressed people, specifically those with bone marrow transplants, have been found to have resistance to the drug foscarnet. However, few studies have described HSV isolates that are resistant to cidofovir. In one instance, cidofovir was used as a treatment for three bone marrow transplant patients who yet displayed signs of an HSV-related condition. One of the three cases was proven to be resistant to cidofovir, and the isolate displayed alterations that reduced the viral DNA polymerase C-terminal length [109]. Notably, cidofovir resistance has also been recorded in children, namely, in three patients with hematopoietic stem cell transplants who received acyclovir and cidofovir in combination as prophylaxis since ganciclovir had negative side effects. Unfortunately, throughout their cidofovir treatment, these children had HSV-related stomatitis, and the investigators speculated that the medication did not stop the patients’ HSV-1 from reactivation [110]. However, cidofovir is regarded as a useful alternative for dealing with HSV isolates that exhibit decreased levels of enzymes involved in the phosphorylation of nucleoside analogues or foscarnet resistance [111]. In a clinical case of a child with HSV-1 that was resistant to ACV and foscarnet, cidofovir therapy was successful in treating this drug-resistant HSV-1 [112]. Other research found that only cidofovir therapy was effective in preventing recurrent oral stomatitis in a girl with lymphatic leukaemia who had HSV-1 isolates resistant to both ACV and foscarnet [113]. New therapeutic options have arisen in recent years as the medications indicated above have considerably diminished clinical efficacy in the treatment of HSV-induced skin lesions. Acyclovir and hydrocortisone for topical application are two of these substitutes (Xerese^®^, Medivir), Huddinge, Sweden. In comparison to acyclovir, this formulation lowered the size of the lesion area by 50% and shortened the duration of herpetic lesions by 1.6 days [114].

Moreover, formulations like docosanol, Viroxyn^®^, and Novitra^®^ attract a lot of attention in the market for showing effectiveness against viral infection caused by HSV [115,116,117].

Docosanol 10% cream, which is easily accessible over the counter (OTC) to treat herpes labialis, is a relatively new medication to treat skin lesions brought on by the virus. It is packaged as a topical cream (Abreva^®^, Avanir, San Diego, CA, USA) [115]. According to one study, using docosanol 10% cream for herpes labialis shortened recovery time by 18 h [118]. Docosanol’s mechanism of action is carried out through the prevention of viral fusion with cell membranes [119]. Viroxyn^®^ (Quadex Pharmaceuticals, UT, USA) is another medication that is marketed to treat herpes labialis. It contains benzalkonium chloride, a Category III antiseptic that is also used for other uses, primarily as a biocidal preservative. According to a study, benzalkonium chloride has virucidal properties against HSV [116]. However, the FDA declared in 2016 that it would no longer allow the sale of several bactericidal ingredients, putting benzalkonium chloride on “stand-by” while awaiting clinical data demonstrating its safety in humans. As a result, it may be recalled [120]. Finally, Novitra^®^, a zinc oxide-based cream, is another drug promoted for treating skin lesions brought on by the HSV-1 virus. In a clinical trial, this formulation has been proven to shorten HSV-1 skin lesions by up to 1.5 days as compared to untreated individuals [117].

### 3.2. Plant-Based Therapeutics

Antiviral medications are used to reduce and lower the intensity of recurring outbreaks of oral and genital herpes caused by HSV-1 and HSV-2 [121]. The major antivirals mentioned above are used for the treatment of oral and genital herpes viral infections [122]. However, conflicting data supporting the use of acyclovir and other nucleoside analogues to treat herpes infection is also present [94,123,124]. Some other antiherpes medicines such as penciclovir and famciclovir work by interfering with viral DNA polymerase but have some inconsistent results [93,94]. Consequently, there is a great necessity to identify new alternative antiviral substances that are derived from plants or natural compounds and demonstrate antiviral efficacy against HSV strains. Interestingly, several plant extracts and phytocompounds derived from plants have been shown to prevent HSV multiplication. 

#### 3.2.1. Plant Extracts 

In the search for novel molecules against HSV infection, plant extracts have drawn a lot of attention. The in vitro and in vivo studies that have been conducted so far on plant extracts were compiled by Garber A et al. [125]. A cell culture study on ethanolic extract of *Eucalyptus camaldulensis* has shown the effectiveness of plant leaf extract against HSV-1 and HSV-2 infection [126]. Alternatively, extracts from *Aglaia odoata*, *Moringa oleifera*, and *Ventilago denticulate* have also been demonstrated to exhibit antiviral activity against wild-type and drug-resistant HSV-1 isolates; however, the mechanisms of action seem to be unknown or not documented [127]. A Chinese herbal plant, namely, *Houttuynia cordata,* prevents HSV-2 infection by preventing NF-κB activation, an important target that has been suggested to be necessary for HSV infection effectiveness [128]. The antiviral efficacy of aqueous and hydroalcoholic extracts obtained from native Chilean plants against HSV-1 and HSV-2 has also been studied. The IC_50_ value of hydroalcoholic extract of *Cassia stipulacea* (80 μg/mL) and *Escallonia illintia* (40 μg/mL) revealed potential antiviral activity against HSV-1. Furthermore, *Aristotelia chilensis*, *Drymis winteri,* and *Elytropus chilensis* also produced satisfactory results against HSV-2 [129]. According to Roner et al. (2007), aqueous extracts of a Chilean soapbark tree, *Quillaja saponaria*, have antiviral activity against HSV-1 and other viruses [130]. Animal models of plant extract also suggest antiviral efficacy against HSV-1 and HSV-2. A study includes the combination of plant extracts and acyclovir to treat antiherpes activity upon infection of the skin in a BALB/c mice model [127] 

#### 3.2.2. Phytocompounds

Eucalyptus species are well-known for their antiviral activity. The main natural compounds present in eucalyptus oil are cineole, alpha-pinene, and others. In a study, 24 compounds were isolated from the leaves of *Eucalyptus sideroxylon* using UPLC/PDA/ESIqTOF-MS method and evaluated for antiviral activity. Out of all the compounds, four compounds were found effective against HSV-1 and HSV-2 [131]. Similarly, it was shown that 12 compounds isolated from *E. globulus* also have proven antiviral activity against HSV-1. The compounds, namely, Tereticornate A and Cypellocarpin C, had potent antiviral activity against HSV-1 and HSV-2, respectively, as compared to acyclovir [132]. The leaves of the plant *Morus alba* have also shown anti-HSV activity, and an active compound present in this plant known as Kuwanon X, a stilbene polyphenol derivative exhibited antiviral activity against different strains of HSV [133]. In addition, some important flavonoids present in *Houttuynia cordata* such as quercetin, quercitrin, and isoquercetin demonstrate stronger inhibition towards HSV-2 [128]. A plant flavonoid, resveratrol, obtained from *Veratum grandiflorum* prevents activation of NF-κB [134]. Animal models and cell culture studies have also confirmed antiviral activity of resveratrol against HSV-1 and HSV-2 [135,136]. Two major compounds isolated, namely, Chikusetsusaponin Iva and calenduloside from the plant *Alternanthera philoxeroides*, were assessed for antiviral activity against HSV-1 and HSV-2. The mode of action of Chikusetsusaponin Iva was found to be virucidal action while evaluating through animal model [137]. Furthermore, it has been revealed that a glycopeptide meliacine (MA) from the plant *Melia azedarach,* shows antiviral efficacy against HSV-1 [138]. Topical application of MA shows antiviral activity against HSV-2 in mouse model [139]

#### 3.2.3. Essential Oils

It has been demonstrated that essential oils derived from plants of the family Labiatae and Verbenaceae have shown antiviral action against HSV. A study conducted on Vero cells against HSV infection has found that in the first phases of infection, essential oils from the Labiatae have shown inhibitory action with *Rf* values of 1 × 10^2^ and 1 × 10^3^ [140]. The essential oil derived from *Glechon spathulata* and *Glechon marifolia* also showed effectiveness against HSV infection [141]. In another investigation, tea tree oil and eucalyptus oil were examined for antiviral efficacy against HSV-1 and HSV-2 using RC-37 cells. Tea tree oil showed a high virucidal effect as compared to eucalyptus oil in the viral suspension test [142]. Volatile oils from *Melissa officinalis*, often known as lemon palm, a member of the Laminae family, have demonstrated antiherpes virus action [143]. A similar study showed the effectiveness of these essential oils against HSV-2 [144]

### 3.3. Algae-Based Therapeutics

Numerous studies have shown that algae contain bioactive compounds with potent antiviral activity against a range of viruses like human papillomavirus (HPV) [145], picornavirus [146], HIV [147,148], influenza [149], and dengue [150]. Additionally, multiple research projects have shown that algae have antiviral action against HSV-1 and HSV-2 [122,151,152]. Four of the more than 36 species of algae collected from Brazil’s beaches were shown to have strong antiviral activity against both HSV-1 and HSV-2. *Stypopodium zonale*, *Ulva fasciata*, and *Codium decorticatum* are some of the examples of green algae that showed antiviral activity against HSV-1 and HSV-2. *Laurencia dendroidea*, a red alga, also showed potential action against HSV-1 which may be due to the presence of sesquiterpenes [152]. According to research, the alga *Padina pavonia*, which also prevents HSV-1 reproduction, has a bioactive substance made of sulphated polysaccharides called fucoidan which prevents the virus from adhering to the surface of cells. An in vivo study on the plant fucoidan represented a decrease in herpetic lesions in the HSV-1 corneal-infected animals who received the compound pretreatments for a week. The same compound obtained from the brown alga *Undaria pinnatifida* also exhibited antiviral properties against HSV-1 and HSV-2 [153]. One more study confirms the antiherpes activity of fucoidan which was isolated from the brown seaweed *Nizamuddinia zanardinii*. The cell line activity of the compound demonstrated that this compound prevented HSV-2 from adhering to Vero cells, hence preventing the first stage of HSV-2 infection [154]. 

It has been observed that polysaccharides from the various algae represent anti-HSV activity. For instance, *Eucheuma gelatinae*, a red alga, has been investigated using Vero cells. The in vitro activity of polysaccharides derived from this alga demonstrates antiviral activity against HSV. It was clear by analysing the expression of the viral protein VP5 as well as the cellular location of this protein that viral protein synthesis was impacted [155]. Another polysaccharide isolated from the green seaweed *E. compressa* exhibits antiviral action against HSV-1 infection. Plaque reduction assays were performed to evaluate the antiherpes activity of these polysaccharides after viral penetration [156]. In another study, polysaccharides isolated from brown alga *Sargassum henslowianum* were purified and subjected to plaque reduction assay. The results confirmed the antiviral activity of polysaccharides SHAP-1 and SHAP-2 with IC_50_ values of 0.89 and 0.82 g/mL, respectively, against HSV-1 [157].

*Osmundaria obtusiloba*, an alga collected from the Brazilian shore, was shown to exhibit activity against both HSV-1 and HSV-2 by interacting with viral glycoproteins [158]. *Hypnea musciformis*, a red seaweed, is another example of an alga having antiviral activities against HSV. It has demonstrated high antiviral activity against HSV-1 in various aqueous fractions. Virucidal activity and the prevention of viral binding to cells are the main mechanisms [159]. Additionally, *Haematococcus pluvialis* extracts have demonstrated antiherpetic efficacy. The major mechanisms were found as reduced virus attachment to the host cell, the viral-cell fusion process, and/or the virus’s entrance into the cell [160]. *Cystoseira myrica* is another source of alga extract with antiviral action against HSV-1, and it significantly reduces the virus’s capacity to replicate [161].

### 3.4. Fungi-Based Therapeutics

In order to find new molecules with antiviral activity against HSV-1 and HSV-2, substances produced from fungi have also been investigated. Polysaccharides-derived *Agaricus brasiliensis* have shown antiherpetic activity against HSV-1 and HSV-2 The cell line study using Vero cells demonstrate a synergistic effect of polysaccharides with acyclovir [162]. It is interesting to note that proteins from fungi also have been found to prevent HSV infection. Two proteins from the fungus *Ganoderma lucidum* that bind polysaccharides were discovered to have antiviral properties against HSV-1 and HSV-2. The neutral protein bound to polysaccharide (NPBP) and the acidic protein bound to polysaccharide (APBP), respectively, prevented plaque formation by HSV-1 and HSV-2. However, APBP has more antiviral activity than NPBP. It is further noted that APBP inhibits virus’s attachment and penetration into Vero cells [163]. The in vitro activity of three anthraquinones reported from the fungus *Aspergillus versicolor* exhibits antiviral activities against HSV-1 [164]. Two secondary metabolites from another species, *Aspergillus ruber* fungus, flavoglaucin, and isodihydroauroglaucin, were also investigated and showed anti-HSV-1 action [165]. Moreover, it was shown that an aqueous extract from the plant *Inonotus obliquus* (AEIO) prevented HSV-1 infection in Vero cells. Further, the antiviral activity of the plant extract suggests prevention of virus entry and membrane fusion. The RC38 protein from *Rozites caperata* also inhibited the growth of HSV-1 in Vero cells and reduced the severity of stromal keratitis in animal models [166]. In contrast, proteins from *Grifola frondosa* (GFAHP) were shown to exhibit antiviral activity against HSV-1 by reducing viral entrance into Vero cells and thus showing virucidal effects. In animal models, GFAHP proteins cause reduction in viral replication, particularly in the cornea, along with vascularization, and thus indicated antiviral efficacy against HSV-1 [167].

### 3.5. Nanotechnology-Based Therapeutics

Nanotechnology offers innovative techniques to create antiviral therapies. The significance of nanotech-derived delivery systems is rising for demonstrating critical pharmacological qualities of bioavailability, faster circulation times, and site-specificity, as well as a reduction in the overall toxicity profile [168,169,170]. Acyclovir was delivered via chitosan nanospheres (NS), a novel topical formulation. The developed nanospheres showed much greater antiviral efficacy against HSV-1 and HSV-2 as compared to acyclovir alone [171]. To enhance systemic bioavailability of buccal films, acyclovir-loaded nanospheres were placed into the films. A significantly substantial increase in acyclovir absorption relative to oral dosage was seen in in vivo testing of the prepared films. Additionally, extended circulation times of the produced film showed that acyclovir continued to release over time [172].

Metal and inorganic nanoparticles (NPs) prevent viral multiplication at the subcellular level, and thus prevent viral invasion. Silver and gold nanoparticles represent significant antiviral action against viruses. Recent research findings have indicated that metal NPs have an anti-HSV-1 action [173,174]. 

The silver nanoparticles (AgNPs) of aqueous extract from the leaves of *Melaleuca alternifolia* exhibit strong antiviral activity against HSV-1, HSV-2 and many other microbes [175]. A hydrogel prepared by tannic acid and silver nanoparticles (TA-AgNP) showed significant capacity to limit HSV-1 infection. The findings suggested that this hydrogel reduced viral adherence, invasion, and post-infection dissemination. It has been also reported that TA-AgNP may bind with proline-rich proteins to prevent viral invasion [176]. The aqueous and hexane extract of *Lampranthus coccineus* and *Malephora lutea* were used to synthesize AgNPs. These nanoparticles possess antiviral activities against HSV-1 infection. A further study shows that AgNPs can represent antiviral action which is connected to the virus’s ability to spread more slowly after being blocked by the interaction with viral envelope glycoproteins [177]. Greenly biosynthesized AgNPs from *Oscillatoria* spp. Demonstrate inhibition of HSV-1 replication in a dose-dependent manner. The algae-biosynthesized gold nanoparticles (AuNPs) from *Spirulina platensis* suppressed HSV-1 proliferation in a dose-dependent manner, culminating in a 90% decrease in cytopathic effect (CPE) at 31.25 μL/mL. [178]. The AuNPs of *Sargassum wightii* (Sw-AgNPs) seaweed also could fight HSV1 infection by antiviral action. The CPE reduction approach was used to assess the anti-HSV-1 activity of AuNPs. The cytotoxicity experiment revealed that Sw-AgNPs were safe at concentrations > 25 μL/mL and demonstrated 85.1% cell survival, while the dose-response inhibition assay revealed that Sw-AuNPs decreased HSV-1 CPE at 10 μL/mL [179]. Three different gold nanoparticles, namely, NPAuG1-S2, NPAuG2-S4, and NPAuG3-S8, were evaluated for antiviral activity against HSV-1. The results suggested that these nanoparticles prevented viral entry and thus reduced HSV-1 infection [180]. In one study, nonfunctionalized AuNPs were used for the evaluation of antiviral efficacy. The results showed less cytopathic effect of these nanoparticles in Vero cells against HSV-1 [181].

Recently, gold nanoparticles of lactoferrin (LF-Ag/AuNPs) have been found effective against genital herpes infection caused by HSV-2. LF-Ag/AuNPs significantly outperformed lactoferrin alone at preventing HSV-2 attachment and penetration into human keratinocytes. Moreover, results found lower viral titres, particularly in genital tissues and spinal cord of infected mice. There was a large decrease in B+T, NK, CD8+, and dendritic cells in the vaginal tissues [182]. In female BALB/c mice, intravaginal application of zinc oxide tetrapod nanoparticles (ZOTEN) has been demonstrated to be an efficient HSV-2 genital infection suppressor. The clinical indications of vaginal infection were significantly reduced by ZOTEN’s potent ability to bind HSV-2, and animal mortality was significantly diminished as well [183,184]. 

Dendrimers, polymeric nanostructures with 100 nm-diameter size that can be used to deliver DNA, siRNA, and antiviral medicines, have been reported to have antiviral activity [185]. The dendrimers interact with viral glycoproteins and prevent HSV-1 infection at early stages. The poly(amide)-based dendrimers exhibited a 35% viral suppression, and at the same concentration as peptidodendrimers, exhibit more than 80% viral inhibition at a dosage of 280 nM/mL. This finding demonstrates the antiviral activity of the dendrimer structure, which could be enhanced by including a specific amino acid sequence into its terminal locations [186]. 

### 3.6. Clinical Trial Evidence

In addition to in vitro investigations, a variety of synthetic compounds have been tested in clinical trials to confirm potential new drugs for the treatment of HSV symptoms. Some of these include antiviral drugs such as amenamevir [187], nelfinavir mesylate [188], brincidofovir [189,190], and pritelivir [191]. The clinical investigations of pritelivir [191] (NCT01047540, NCT01658826, and NCT02871492) and brincidofovir (NCT01143181) showed activity in phase III trials and were found effective against HSV infection. However, the findings have not been published. One more clinical study of pritelivir tablets on immunocompromised individuals is currently ongoing for the treatment of acyclovir-resistant HSV-1 or HSV-2 infection. The results of this study are expected in the year 2024 (NCT03073967). It has been further reported that brincidofovir has up to 1000 times the antiviral activity of cidofovir [192]. A clinical trial was conducted to assess the efficacy of topical cidofovir for refractory mucocutaneous HSV-1 and HSV-2 in AIDS; the findings of this clinical trial, however, have not been published (NCT00002116). An oxadiazolephenyl derivative, amenamevir, mainly focused on the viral DNA helicase/primase complex (H/P) [106,193], has undergone evaluation in at least three clinical studies (NCT02209324, NCT01959295, and NCT02852876) [187]. Unfortunately, a study shows that amenamevir had side effects in an early clinical phase [187]. In addition to being an inhibitor of the HIV-1 protease [194,195], the antiviral drug nelfinavir mesylate was also found to have antiviral efficacy against HSV-1 [188]. The clinical study of this antiviral drug is under evaluation for the treatment of Kaposi’s sarcoma and HSV infection (NCT03077451). 

Clinical experiments have been also conducted on several phytocompounds based on the research performed in vivo, in order to verify new medications that might be used to cure HSV infection. Various Chinese herbs including Huangbo (*Cortex Phellodendri*), Zhizi (*Scutellaria baicalensis*), and Huanglian (*Rhizoma coptidis*) have been tested for the treatment of herpes labialis (NCT03469232) A Thera Neem Lip therapy containing numerous active ingredients like coconut oil, sesame oil, jojoba oil, neem oil, peppermint oil, and tocopherol is in clinical trials for the treatment of HSV (NCT00985335). Additionally, clinical research evaluated Viblock^®^, a cream that is allegedly made entirely of botanical ingredients but whose composition has not been disclosed (NCT03080961). The cream’s ability to prevent HSV-2 infection was investigated during formulation. However, the outcomes of the clinical trials mentioned above have not yet been published. In 2019, a randomized controlled clinical trial of medical grade kakua honey was conducted in comparison with acyclovir against herpes labialis. The topical application of natural extract and 5% acyclovir honey was given five times a day, and results found that kakua honey was no more effective than acyclovir 5% [196].

## 4. Limitations of the Current Treatments and Challenges for Vaccine Development

Treatments that are commercially available to treat HSV-1 and HSV-2 infection have some serious side effects, which is a matter of deep concern. Currently, acyclovir and valacyclovir are widely being used for the treatment of HSV-1 and HSV-2 infections. However, certain limitations are also associated with these antiviral drugs [197]. The developing resistance of some antiviral medications such as valacyclovir, penciclovir, and famciclovir in immunocompromised patients is also a major challenge [111,198,199]. Moreover, these antiviral drugs are not able to control latency or latency reactivation processes. Some of the limitations of current therapies are described below.

### 4.1. Limitations of Current Treatments

Current therapies such as acyclovir and valacyclovir, two of the most-often used treatments for HSV, work well to shorten the length and intensity of symptoms as well as viral shedding [197]. However, the short treatment window of HSV infections for effective therapy is limited. Furthermore, they are only marginally helpful, with only slight improvements in symptom intensity and duration [200]. It is noted that oral administration of ACV has an absorption efficiency of just 15–30%, and prior studies suggested that elderly patients with renal issues could develop severe neurotoxicity as a result of their inability to excrete this medication correctly [201,202]. The propensity of HSV-1 and HSV-2 to evolve and create forms that are resistant to this drug by attaining mutations in the gene that encodes for the viral thymidine kinase, as well as the gene that encodes for viral DNA polymerase (UL30), is another significant drawback of ACV [203]. However, it is possible for patients to develop resistance to valacyclovir, penciclovir, and famciclovir. As valacyclovir is derived from acyclovir, there are chances of development of cross-resistance between valacyclovir- and acyclovir-resistant HSV isolates [111,198]. Acyclovir-resistant HSV-1 isolates in immunocompromised individuals, on the other hand, may develop cross-resistance to penciclovir and the prodrug famciclovir [199,204,205,206]. If administered as soon as prodrome or erythema symptoms are noticed, oral ACV treatment for skin lesions caused by HSV-1 and HSV-2 only shortens the recovery process by around 2 days (time to disappearance of scab), from 7.9 days (placebo arm) to 5.8 days [207]. The therapeutic value of these antiviral medications for the treatment of herpetic lesions has been somewhat disputed because these changes are mild yet statistically significant [208]. Importantly, a substantial impact for ACV is not shown when the medication is administered at the stage of papule [208]. Additionally, in this case, it was discovered that the treatment group required longer time for cutaneous lesions to heal than the placebo arm did (8 days vs. 7.2 days, respectively). Unfortunately, over 50% of patients are unable to recognise the prodrome and erythema stages before papule development, and as a result, they are not able to begin a successful oral therapy with ACV in time to considerably shorten the duration of the herpetic lesions [208]. ACV therapy is thus not beneficial in these situations [209]. ACV can be used topically as a cream instead of being consumed orally. However, topical administration of ACV to herpetic lesions at the papule stage has therapeutic effects, which are generally modest as they only shorten the recovery period in *herpes labialis* by one to two days and in genital herpes infections by three days [10,11]. It should be noted that ganciclovir has been reported to cause significant negative side effects in an elevated proportion of individuals, including nephrotoxicity, myelosuppression, neutropenia, confusion, anxiety, altered mental status, ataxia, convulsions, tremors, fever, abnormal liver enzyme levels in serum, nausea, and diarrhoea [96]. Cidofovir and foscarnet are also associated with a variety of negative side effects in patient populations, including nephrotoxicity, azotemia, proteinuria, crystalluria, interstitial nephritis, acute tubular necrosis, rise in creatinine levels of up to 50%, hypo- and hypercalcemia, hypo- and hyperphosphatemia, and the development of urogenital ulcers [210]. Due to these side effects, it is advised that foscarnet patients be closely monitored to prevent the anomalies mentioned above. Foscarnet resistance, unlike that of nucleoside analogues, is exclusively brought on by alterations in the viral DNA polymerase gene. Immunosuppressed people, specifically those who have bone marrow transplants, have been found to have resistance to the drug foscarnet [111].

### 4.2. Various Approaches Investigated for Meeting out the Limitations and Challenges

The incidence and prevalence of HSV-2 infection at the community level are unlikely to be impacted by current HSV preventive or treatment methods. Therefore, HSV vaccines may be the only practical and approachable means of achieving this objective. Even though currently there is no vaccine for herpes labialis and genital herpes, there are a number of candidates in clinical and pre-clinical stages of research. There are two main areas of vaccine development: preventive and therapeutic, with some having dual purposes [103,200]. The main goal of preventative vaccination is to keep a seronegative individual from developing a primary infection. Therapeutic immunizations are intended to stop HSV reactivation, lessen the frequency of relapses, or lessen the intensity or duration of clinical symptoms [211]. In terms of vaccine development, a successful vaccination would most likely induce both humoral and cell-mediated responses. In each vaccination study, it is critical to pay attention to the adjuvants and assess how they contribute to the development of a robust humoral and cell-mediated response [212]. 

## 5. Preventive Methods for HSV through Vaccines

To combat HSV-1 and HSV-2 infections, four main vaccine strategies have been developed, including replication-defective HSV vaccines, inactivated killed HSV vaccines, live-attenuated HSV vaccines, and subunit HSV vaccines. In terms of safety and efficacy, these vaccine strategies have various advantages and disadvantages. 

### 5.1. Inactivated Killed HSV Vaccines

In the 1970s and 1980s, an approach to kill the whole virus led to the development of the first inactivated killed HSV vaccine [213,214]. Since these inactivated HSV vaccines produced only antibodies instead of T cells, they were ineffective at preventing recurring HSV-1 or HSV-2 infections and illnesses [214,215,216]. The ineffectiveness of inactivated viral vaccines has been overcome by live-attenuated viral vaccines by showing their ability to replicate. These two vaccines vary in important ways; the inactivated vaccines have the potential to modify the antigens while the antigens produced by live-attenuated vaccines retain their native structure, thus increasing vaccine effectiveness. In contrast to live-attenuated vaccines, inactivated vaccines stimulate the body to produce antibodies, but the immunological response is significantly delayed [217,218]. Additionally, as is the case with the poliomyelitis vaccines, inactivated and live-attenuated vaccines can be administered through several routes against a specific type of virus. Thus, it is impossible to say with certainty that live-attenuated vaccines are better than inactivated vaccines due to their persisting capacity for replication [217,219,220].

### 5.2. Live-Attenuated Vaccines

Over the past 24 years, numerous live-attenuated HSV vaccines have been developed and evaluated in animal models mostly as preventative therapy. However, only a handful of these live vaccines have advanced into clinical investigations due to tolerability and safety issues [221]. For the purpose of creating in vitro viral stocks, the mutant HSV-2 gD/+gD-1 virus needs a gD1-complementing cell line. The gD-2 virus can only replicate itself in vivo for one replication cycle. It was established that this vaccine’s protection was brought about through antibody-dependent cell-mediated cytotoxicity (ADCC). Following intravaginal injection, this vaccine provided protection against genital disease and decreased skin lesions and inflammation in mice [222,223]. The vaccine offered defence against several clinical isolates, some of which originated in Africa, as well as laboratory strains of HSV-1 and HSV-2 [222]. Another live-attenuated vaccine, AD472, had been tested in guinea pigs in the year 2005 [224]. The guinea pig genital HSV model was also employed in early tests of R2 to assess its capacity to reproduce at the site of injection, induce illness, and infect brain tissues. The results indicated good efficacy of R2 vaccine when administered through an intradermal route [225]. The effectiveness of herpes vaccination is frequently investigated using female inbred mice in case of genital herpes infection [226]. These mice are widely accessible, simple to keep, and have transgenic or knockout strains to study defence systems [227]. Recent investigations in a murine model have demonstrated that both HSV-1 seronegative and seropositive animals challenged with HSV-2 did not develop latent infection after receiving the gD-2 vaccine [228].

### 5.3. Replication-Defective Virus Vaccines 

Also known as disabled infectious single cycle virus vaccines, these contain one or more dysfunctional genes that are crucial for the replication of the viral genome. Immune responses can be induced by disabled infectious single cycle virus vaccines, but no viral offspring are created. These vaccines are less immunogenic since they have reduced capacity to elicit antigens presenting cells such as dendritic cells and macrophages, which are necessary to produce CD4+ and CD8+ T cell responses. In 1996, McLean evaluated DISC HSV-1 vaccine in guinea pigs [229]. Later, gH-deleted HSV-2 mutant was evaluated in guinea pigs for recurrent genital herpes infection by the same researcher [230]. 

The HSV-2 dl5-29 (HSV529) vaccine, which has been the subject of the most research, was created by Knipe in 2008 and other researchers in 2010 [215,221,231]. HSV529 has mutations in the vital viral genes UL5 and UL29 that prevent replication. Both a preventive and a therapeutic vaccine were attempted. A trial demonstrated safety of HSV529 by inducing CD4+ T-cell responses and neutralising antibodies in seronegative individuals who received the vaccine [232]. Recent research has shown that the generation of antibodies mediates the activation of NK cells. Moreover, the cervicovaginal fluid contained HSV-2 gD antibodies at a level about one-third that of serum [233]. Table 2 includes vaccines in different phases of trials. 

### 5.4. Subunit HSV Vaccines 

Despite being less dangerous than live-attenuated vaccines, the development of subunit vaccines faces difficulties in producing an immune response that is both efficient and long-lasting. Because of this, adjuvants are frequently used while administering subunit vaccines [267]. Proteins, DNA, and peptide epitope-based vaccines are a few of the several subunit HSV vaccination strategies that have been explored [259,263]. The first therapeutic vaccination experiment was conducted in 1994. In this study, 98 symptomatic genital herpes patients were selected who experienced four to fourteen recurrences annually; they received the gD vaccine with aluminium salt (often known as “Alum”) adjuvant (96). Regrettably, while the vaccine was able to increase neutralising antibodies to HSV-2 fourfold over baseline levels, this vaccine only managed to lower the prevalence of HSV-infection by 24% [268]. The GlaxoSmithKline glycoprotein D2 vaccine is a subunit vaccine made of the HSV-2 glycoprotein D (gD2-AS04) with adjuvants such as aluminium hydroxide and 3-O-deacylated monophosphoryl lipid A (MPL) [237,246,247,252].

A subunit vaccine, HerpV, consisting of 32 HSV-2 peptides complexed with Hsc70 human recombinant protein has shown significant CD4+ and CD8+ T cell responses in HSV-2-positive healthy volunteers. Previous in vivo models demonstrated protection from viral assaults. Mononuclear cell reactivity and CD8+ T-cell proliferation were seen in recent clinical trial results from HSV-2+ patients who received the vaccine [248,249]. Another subunit vaccine, GEN-003, consist of ICP4 and gD2 truncated proteins that are further combined with a novel adjuvant, Matrix M2 (MM-2) [259]. This saponin adjuvant shows immunostimulatory effect for both B and T cells. This trial significantly reduces the viral shedding and recurrent lesions of herpes. This defence seems to be linked to antiviral CD4+ and CD8+ T cell responses produced from blood [256,257,258]. A subunit trivalent vaccine, namely, gC2/gD2/gE2 with a CpG/aluminium adjuvant contains three main glycoproteins, namely, C, D, and E. These glycoproteins are immune-resistant molecules. According to research done on rhesus macaques, the trivalent vaccine can produce neutralising antibodies which prevent the action of gC2 and gE2 immune evasion but induced CD4+ T-cell responses. The guinea pig model was used to assess the vaccine’s effectiveness, and it was discovered to be extremely effective in lowering the severity and frequency of genital lesions [252,253]. Recombinant HSV-2 proteins gD, UL19, and UL25 gene products, namely, G103, together with a TLR4 agonist adjuvant demonstrated full protection against deadly HSV-2 infection in HSV-2-infected mice. An increase in CD4 and CD8 T cells is also reported in the study. A 50% reduction has been observed in the number of lesions in a guinea pig model [226].

### 5.5. Nucleic Acid Vaccines

Two primary types of nucleic acid vaccines are DNA plasmid vaccines and mRNA vaccines. These vaccines are designed to deliver genetic information to cells so that they may express and synthesize proteins. These proteins act as antigens for the body’s immune system. Recently, mRNA technology has advanced substantially, offering important benefits. It exhibits rapid protein synthesis for a controlled period, does not integrate into the host genome, and can be expressed in both proliferating and non-proliferating cells [269]. A DNA vaccine named COR-1 showed humoral and cellular responses along with decreased viral latency and protection from fatal virus challenge. Two main codons, such as HSV-2 envelope gD2 and the shorter version of gD2, are linked with COR-1 [261]. The clinical study states a reduction in viral shedding; thus, the vaccine was considered to be safe for HSV-infected people [260]. The immune responses for the management and prevention of HSV infection may be different from each other. For controlling recurrent herpes, CD8+ T lymphocytes are very essential. Consequently, recurrent genital herpes therapy antigens may be different from those used for prevention [270,271,272]. 

## 6. Conclusions 

The wide variety of antiherpetic medicines are known only for shortening the length and severity of symptoms as well as virus shedding, but that too in the later stage. For best results in limiting viral replication, therapy must be initiated during the febrile phase as late treatment is ineffective. It also has been reported that inhibition therapies are only 50% effective in recurrent infections [9]. Increasing resistance and side effects of FDA-approved drugs such as acyclovir and foscarnet also limit the use of synthetic drugs [200]. The cell culture model and animal studies state that various plant, fungus, and algae-derived substances have been shown to have potent antiviral effects against HSV-1 and HSV-2. It has been reported that various plant extracts and bioactive compounds exert antiviral properties [273]. Some important modes of action of these natural compounds include prevention of virus entrance, replication, and viral protein expression [274]. However, certain side effects have also been reported of these natural medicines in comparison to others [275,276,277,278]. 

The complexity of HSV pathophysiology and immune invasion is continually being studied. Considering the widespread distribution of HSV, several attempts have been made to develop an effective vaccine. Existing vaccine candidates for HSV are not very successful in clinical trials. Thus, combinatorial vaccines appear to be the most suited vaccines. The major aim of developing a vaccine is to prevent viral transmission and lessen severity of disease. However, understanding these mechanisms in more detail is necessary in order to create a therapeutic or preventive vaccine. 

It is anticipated that this review will have helped to highlight significant gaps in the available therapeutic options, which are essential to understand for the development of a successful and novel HSV vaccine.

## Figures and Tables

**Table 1:** Potential targets for HSV prevention.

Mechanism of Action	Potential Target	Drugs	References
Suppresses herpes virus genome replication at the protein level	DNA polymerase	4-hydroxy-quinoline-3-carboxamide molecule and 8-hydroxyquinoline.	[52,53]
Blocks attachments and invasion by targeting viral proteins	Glycoproteins	PRO 2000, polymethylene hydroquinone sulfonate, and cellulose sulphate	[55,56]
Inhibits HSV DNA replication	Helicase primase complex	2-amino-thiazole and thiazolylsulfonamide	[57]
Ribonucleotide reductase inhibitors	Ribonucleotide reductase	Peptidomimetic inhibitors	[58,59]
Inhibits polyamine biosynthetic pathway to block replication of HSV	Polyamine pathway	SAMDC inhibitors	[63,64]
CDK-2 inhibitors	CDK-2	Roscovitine	[65,66]
Blocks attachment and penetration	Entry receptors (HVEM, glycosaminoglycans)	Anti-HVEM antibodies	[54]
TLR inhibitors	TLR	G-ODN, CpG oligonucleotide	[67]

**Table 2:** Vaccination strategies for the prevention of HSV-1 and HSV-2 infection.

Sr. No.	Type of Vaccine	Candidate Name	Modification in Vaccine	Host	Phase	Results	Origin	Refs.
1	Inactivated vaccine	HSV-2 dl5-29	deletions of UL5 and UL29	mice and guinea pigs	Pre-clinical	enhance immune responses, protection against reactivates HSV infection	Harvard Medical School, Boston	[234]
2	Inactivated vaccine	SC16ΔgH	deletion of HSV-1 gH deletion	mice	Clinical	has no impact on viral shedding and inability to demonstrate immunity against reactivated genital herpes infections	Boston, Massachusetts	[16]
3	Replication-Defective Viral Vaccines	CJ2-gD2	Dominant negative HSV-2 with CJ2-gD2	guinea pigs	Pre-Clinical	provide protection against primary infection and reactivates HSV-2 genital infections	-	[235]
4	Replication-Defective Viral Vaccines	HSV-1 vhs-/ICP8-	deletions of vhs in an ICP8	mice	Pre-Clinical	significantly enhances protective efficacy	-	[236]
		Δ41Δ29	deletions in the gene for virion host shutoff (vhs) protein	BALB/c mice	Pre-Clinical	decreased the frequency of UV-B-induced recurrent viral shedding in mice with latent infection	Washington University School of Medicine	[237]
5	Replication-Defective Viral Vaccines	HSV529	Deletion of UL5, UL29 in HSV-2 *dl5-29* mice	mice and HSV-1 seropositive guinea pigs		immunogenicity reported	Sanofi Pasteur	[238]
		HSV529	-	human participants	Phase I	induced moderate CD4+ T-cell responses and neutralising antibodies in HSV-seronegative vaccine recipients	Sanofi Pasteur	[232,233]
6	Live-attenuated vaccine	HSV-2 ΔgD2	Deletion of gD-2	C57BL/6 or BALB/c mice	Pre-clinical	protection by non-neutralising Fc-mediated humoral responses	Albert Einstein College of Medicine	[222]
				C57BL/6 mice	Pre-clinical	infected mice were protected by gD-2	Albert Einstein College of Medicine	[223]
7	Live-attenuated vaccine	AD472	Deletion of γ134.5 gene, UL55-56, UL43.5 and the US10-12 region	guinea pigs	Pre-clinical	reduce lesion development and infection severity in guinea pigs	Albert Einstein College of Medicine	[224]
8	Live-attenuated vaccine	NE-HSV-2	Antigens such as gB2 and gD2 were used to make nanoemulsion	C57BL/6 mice	Pre-Clinical	reduction of reactivated lesions and viral shedding by more than 50%	BlueWillow Biologics	[239]
9	Live-attenuated vaccine	R2	Mutated pUL37 gene in the R2 region	guinea pigs	Pre-Clinical	reduction of reactivated virus shedding	Thyreos LLC	[225,240]
10	Live-attenuated vaccine	HSV-1 VC2	Mutations in glycoprotein K (gK) and UL20	rhesus macaques	Pre-Clinical	induction of immune response	Louisiana State University	[241]
			-	guinea pigs	Pre-Clinical	diminishes HSV-2 replication	Louisiana State University	[242]
			Mutations in glycoprotein K (gK) and UL20	mice	Pre-Clinical	protect mice against lethal intravaginal infection	Louisiana State University	[243]
11	Live-attenuated vaccine	HSV-2 0ΔNLS	Deletion of *ICP0*^−^	mice	Phase II	represent avirulence and immunogenicity	Southern Illinois University	[244]
					Pre-Clinical	protected against lethal challenge with wild-type HSV-2	Southern Illinois University	[245]
12	Subunit Vaccine	gD2 subunit vaccine	gD2 adjuvant with AS04	HSV-1– and HSV-2–seronegative women	Phase 3	efficacy against HSV-1 and HSV-1 genital disease was 58% and 35% respectively	GlaxoSmithKline	[246]
				girls aged 10–17 years	Phase 3	vaccine was tolerated and immunogenic	GlaxoSmithKline	[247]
13	Subunit Vaccine	HerpV	32 synthetic 35mer HSV-2 peptides complexed with Hsc70 protein + QS21 (saponin adjuvant)	HLA-A2 transgenic mice	Pre-clinical	immunogenic, CD4(+) and CD8(+) T cell activators	Agenus	[248]
			HerpV+ QS-21	human participant	Phase II (DC)	significant CD4+ T and CD8(+) T cell response	Agenus	[249]
14	Subunit Vaccine	HSV-2 trivalent vaccine	Combination of glycoproteins C, D, and E (gC2, gD2, gE2)	guinea pig	Pre-clinical	trivalent protein vaccine provides protection to prevent herpes infection	University of Pennsylvania	[250,251,252]
				rhesus macaques and guinea pig	Pre-clinical	viral shedding was reduced	University of Pennsylvania	[253]
				guinea pigs	Pre-clinical	immune evasion domains on HSV-2 glycoproteins	University of Pennsylvania	[254]
15	Subunit Vaccine	HSV1 gB lentiviral vector	HSV-1 glycoprotein B (gB1) with feline immunodeficiency virus (FIV) vector	C57BL/6 mice	Pre-Clinical	significant reduction in viral infectivity	University of Pisa, Italy	[255]
16	Subunit Vaccine	GEN-003	gD2ΔTMR + M2	seropositive participants	Completed Phase II (DC)	reduction in shedding and lesion rate was reported	University of Cincinnati,	[256]
			gD2 (truncated)+ICP4 fragment	healthy participants	Phase 2	reduction in shedding rate and lesion rate	Genocea	[257,258]
			GEN-003/MM-2	guinea pigs	Pre-clinical	elicits humoral immune responses, CD4+ and CD8+ T cells	Genocea	[259]
17	Subunit Vaccine	G103	HSV-2 gD, deletions in UL19 and UL25	Mice and guinea pigs	Pre-clinical	reductions of no. of lesions and lesion area	Immune Design	[226]
18	DNA Vaccines	COR-1	gD2 ubiquitin tag with COR-1 DNA or placebo	HSV-2 seropositive subjects	Phase II	reduction in viral shedding	Admedus	[260]
				BALB/c mice	Pre-clinical	protects against lethal viral challenge and reduces ganglionic latency	University of Washington	[261]
19	DNA Vaccines	pRSC-gD-IL-2123	HSV-1 gD combined with IL-21	mice	Pre-clinical	inhibition of HSK in HSV-1-infected mice	Southeast University, China	[262]
20	DNA Vaccines	Vaxfectin^®^-gD2/UL46/UL47	HSV-2 glycoprotein D and UL46 and UL47 genes/Vaxfectin	mice	Pre-clinical	reduction of frequency of reactivated disease and viral shedding	Vical	[263,264]
		Vaxfectin-gD2 pDNA	pDNA + FL gD2 Vaxfectin	guinea pig	Pre-clinical	reduction in HSV-2 DNA copy number	University of Washington	[265]
21	DNA Vaccines	gB1s-NISV	NISV+HSV-1 gB+CpG	mice	Pre-Clinical	provides protection against a heterologous lethal vaginal challenge with HSV-2	-	[266]

## Data Availability

Not applicable.
